# Australian Paediatric Acute Code Stroke study: study protocol for a quasi-experimental trial measuring the impact of Code Stroke protocols on time to diagnosis and access to reperfusion therapies

**DOI:** 10.1136/bmjno-2026-001582

**Published:** 2026-09-03

**Authors:** Mark T Mackay, Belinda Stojanovski, Nicola Fearn, Michael Fahey, Ian Andrews, Christopher Troedson, Richard Webster, Christina Miteff, Adriane Sinclair, Claire Pridmore, Snehal Shah, Claire G Spooner, Leonid Churilov, Bruce C V Campbell, Lan Gao, Dominique Cadilhac, Franz E Babl, Dee Banks, Lisa Murphy, Associate Investigators, Bruce Campbell

**Affiliations:** 1 Neurology, Royal Children’s Hospital, Melbourne, Victoria, Australia; 2 Clinical Sciences, Murdoch Children’s Research Institute, Melbourne, Victoria, Australia; 3 Neurology, The Royal Children’s Hospital Melbourne, Melbourne, Victoria, Australia; 4 Clinical Sciences, Murdoch Children’s Research Institute, Parkville, Victoria, Australia; 5 Paediatrics, University of Melbourne, Melbourne, Victoria, Australia; 6 Paediatrics, Monash University, Melbourne, Victoria, Australia; 7 Neurology, Monash Children’s Hospital, Clayton, Victoria, Australia; 8 Neurology, Sydney Children’s Hospital, Sydney, New South Wales, Australia; 9 Neurology, The Children’s Hospital at Westmead, Sydney, New South Wales, Australia; 10 Neurology, John Hunter Children’s Hospital, Hunter Region Mail Centre, New South Wales, Australia; 11 Neurology, Queensland Children’s Hospital, Brisbane, Queensland, Australia; 12 Neurology, Women’s and Children’s Hospital South Australia, Adelaide, South Australia, Australia; 13 Neurology, Perth Children’s Hospital, Perth, Western Australia, Australia; 14 Paediatrics, University of Western Australia, Perth, Western Australia, Australia; 15 Neurology, Starship Children’s Hospital Child Protection Service, Auckland, New Zealand; 16 Medicine and Neurology, University of Melbourne, Melbourne, Victoria, Australia; 17 Florey Neuroscience Institutes, Parkville, Victoria, Australia; 18 Medicine, University of Melbourne, Parkville, Victoria, Australia; 19 Neurology, Royal Melbourne Hospital, Melbourne, Victoria, Australia; 20 Deakin Health Economics, Institute for Health Transformation, Deakin University Faculty of Health, Burwood, Victoria, Australia; 21 Stroke and Ageing Research, Department of Medicine, School of Clinical Sciences, Monash University, Melbourne, Victoria, Australia; 22 Little Stroke Warriors, Melbourne, Victoria, Australia; 23 Stroke Foundation Australia, Melbourne, Victoria, Australia

**Keywords:** STROKE, PAEDIATRIC NEUROLOGY

## Abstract

**Introduction:**

Childhood stroke leads to long-term neurological impairment, impacts on quality of life and has substantial economic costs. Coordinated systems of care are required to streamline rapid diagnosis and treatment of stroke. The primary aim is to investigate the effect of Code Stroke protocols on increasing the proportion of children with stroke diagnosed within the 4.5-hour time window for reperfusion therapies.

**Methods and analysis:**

Prospective, multicentre, quasi-experimental interventional study in nine Australian and New Zealand children’s hospitals from 2020 to 2026. Children aged 1 month to <18 years with stroke symptoms are eligible for inclusion. A staged pre-implementation and post implementation study design will investigate effects of (1) Code Stroke protocols to streamline diagnosis and treatment, (2) decision support tools to improve diagnostic accuracy and (3) perfusion imaging to quantify brain at risk of infarction. Presenting characteristics, timelines of care, treatments, functional and health economic outcomes will be collected. The primary outcome is the proportion of children with stroke diagnosed within 4.5 hours, estimated using a mixed-effect logistic regression model, with the dichotomous variable time to diagnosis as the dependent variable, implementation phase as the independent fixed effect and hospitals as random effects. Sample size calculation estimates that 98 patients with radiologically confirmed stroke per phase will yield 80% power to detect change from 15% to 40% in the proportion of children diagnosed within 4.5 hours.

**WHAT IS ALREADY KNOWN ON THIS TOPIC**

- Reperfusion therapies including thrombolysis and clot retrieval, which save viable brain, have transformed health outcomes for adults with stroke. Children are not accessing reperfusion therapies because of delays to diagnosis.
- There are limited data reporting efficacy and safety of reperfusion therapies, and their impact on functional outcomes, because major trials have excluded patients younger than 18 years of age.

**WHAT THIS STUDY ADDS**

- This will be the first multicentre, prospective study of acute childhood stroke care undertaken in Australia and New Zealand to improve the understanding of the impact of Code Stroke protocols on improving patient outcomes.
- This pragmatic, real-world study will provide new evidence for the effect of standardised clinical and diagnostic imaging protocols, advanced stroke imaging and decision support tools on the efficiency and effectiveness of stroke diagnosis and treatment practices.

**HOW THIS STUDY MIGHT AFFECT RESEARCH, PRACTICE OR POLICY**

- This study will provide best available data on the effect of interventions to identify children most likely to benefit from reperfusion therapies that can save viable brain.
- This study will have a bi-national impact in Australia and New Zealand and will be relevant for international efforts to improve systems of acute stroke care for children.
- The study will provide data on the impact of shortening time to diagnosis and increased access to reperfusion therapies on functional and health economic outcomes.

## Introduction

 Childhood stroke has a pooled incidence of 2.1 per 100 000 person-years.^1^ Global impact is growing, with a 35% increase in prevalence from 1990 to 2013.^2^ Childhood stroke is associated with high rates of neurological impairment and substantial economic costs.^3 4^ Stroke is eminently treatable but time-critical. Reperfusion therapies, which include intravenous thrombolysis and endovascular clot retrieval (ECR), are established as effective to improve long-term outcomes of adults with acute ischaemic stroke.^5 6^

The catchcry in emergency stroke treatment that *time is brain* is equally applicable to children because failure to save brain results in poorer outcomes.^7^ Survivors of childhood stroke face decades of living with disability and with that comes a significant impact on their families and the broader community. The long-term economic and societal cost of childhood stroke is not well elucidated, with previous US studies reporting the economic impact only up to 5 years from stroke event.^3^

The days of therapeutic nihilism in adult stroke have passed, yet children are still not receiving the benefits of reperfusion therapies, often because of diagnostic delay, sometimes extending beyond 24 hours.^8^ Delays prior to arrival relate to limited awareness of stroke among caregivers,^9^ clinical recognition among paramedics^10^ and emergency physicians,^11^ and poor accuracy and reliability of prehospital decision support tools.^12^ In contrast to adults, in-hospital factors—particularly delayed access to rapid diagnostic imaging in the emergency department (ED)^13^ and poor sensitivity of CT-based imaging for detection of infarction in children with arterial ischaemic stroke (AIS)^14^—are more important contributors to delayed diagnosis than prehospital factors. Therefore, institutional and regional systems of acute care are required to streamline diagnosis and initial treatment of childhood stroke.

In adults with stroke, there has been a move towards tissue-based rather than time-based selection of candidates for reperfusion therapies to salvage viable brain. This approach using advanced brain imaging, which quantifies penumbral tissue destined for infarction if blood-flow is not restored, has led to use of thrombolysis^15 16^ and ECR^17^ in extended time windows beyond 4.5 and 6 hours, respectively. These diagnostic advances have only recently been tested in observational studies in children^18 19^ and require further investigation to determine their utility to identify children most likely to benefit from treatment.

Data from an international registry suggests that thrombolysis is safe in children^20^ but efficacy for improving functional outcomes remains unclear, with the literature mainly confined to retrospective studies^21–26^ reporting outcomes at the time of discharge^21–23 25^ or in combination with ECR at 90 days.^21 24 26 27^ A recently established prospective international registry of 208 paediatric patients provides evidence for efficacy of ECR in children with large or medium vessel occlusion. Despite having more severe stroke symptoms, children treated with ECR had better functional outcomes observed at 90 days post-stroke when compared with those treated with best medical therapy (which included thrombolysis).^27^

Despite these limitations, there has been substantial progress over the past 10 years in the development of clinical practice guidelines^28 29^ and institutional paediatric stroke protocols to standardise the approach to acute recognition, diagnosis and emergency treatment of childhood stroke. Recent studies from France^30^ and Spain^31^ have demonstrated the benefits of developing regional hospital systems of acute care, with designation of hospital-based primary and comprehensive stroke centres. No studies, however, have adopted a coordinated national approach to acute stroke care, with protocols tailored to local resources.

### Objectives and hypotheses

The primary aim of the Australian Paediatric Acute Code Stroke (PACS) study is to investigate the effect of Code Stroke protocols on increasing the proportion of children aged 1 month to less than 18 years of age who are diagnosed within 4.5 hours of stroke symptom onset—as the time window for thrombolysis.

The secondary aims of the study are to (1) increase the proportion of children receiving reperfusion therapies, (2) investigate the feasibility and utility of automated perfusion software to guide clinical decision making, (3) test the utility of clinical decision support tools to improve accuracy of paramedic and ED physician diagnosis, (4) estimate the economic impacts of shorter time to diagnosis and access to reperfusion therapies and (5) benchmark functional outcomes at 3 months post-event against adults with stroke.

The main research questions are: do acute stroke protocols (1) decrease the time to diagnosis of childhood stroke and (2) increase access to reperfusion therapies that salvage viable brain?

We hypothesise that standardised institutional Code Stroke protocols, in combination with automated perfusion imaging and clinical decision support tools, will improve the efficiency (shorter time to diagnosis) and effectiveness (increased access to reperfusion therapies) of acute stroke care in children with stroke.

## Methods and analysis

### Study design

A quasi-experimental, two-tiered preintervention and postintervention study design will be used to evaluate the effects of the study interventions in tertiary children’s hospitals in Australia and New Zealand. The pre-implementation phase is the 3-year period from 2015 until 2017, reflecting the era prior to the launch of the Australian guideline for the acute management and diagnosis of stroke in children.^28^ The combined protocol development and post implementation phases are from 2018 to 2026. The study will follow the Transparent Reporting of Evaluations with Non-randomised Design statement for quasi-experimental non-randomised trials.^32^

### Patient and public involvement

A Steering Committee has been established to oversee governance of the study and reporting of progress, which is chaired by the Stroke Foundation (as the grant recipient and leading non-government stroke advocacy organisation in Australia). The committee has representation from Australian parents with lived experience of childhood stroke, to provide expert knowledge, advice and guidance, and to represent the interests and viewpoints of the Australian and New Zealand childhood stroke community.

### Study setting and procedures

The study is being conducted at eight tertiary paediatric hospitals in Australia and one tertiary paediatric hospital in New Zealand that meet recommended international criteria for primary paediatric stroke centres^28^ from 2020 until 2026. These hospitals include (1) the Royal Children’s (RCH) and (2) the Monash Children’s Hospital (MCH), both in Melbourne, Victoria, Australia, (3) the Sydney Children’s Hospital (SCH), (4) the Children’s Hospital at Westmead (CHW), both in Sydney and (5) the John Hunter Children’s Hospital (JHCH), Newcastle, New South Wales, Australia, (6) the Queensland Children’s Hospital (QCH), Brisbane, Queensland, Australia, (7) the Women’s and Children’s Hospital (WCH), Adelaide, South Australia, (8) the Perth Children’s Hospital (PCH), Perth, Western Australia and (9) the Starship Children’s Hospital (StCH), Te Toka Tumai, Auckland, New Zealand (online supplemental e-Table 1).

The Code Stroke protocols tailored to local resources are being primarily followed in the EDs but also target inpatients with conditions associated with a risk of stroke (eg, children with cardiac disease). The EDs are members of the PREDICT research network.^33^ These hospitals will partner with adult comprehensive stroke centres to develop PACS protocols tailored to each hospital. Common elements will include (1) clinical criteria that trigger activation of the Code Stroke protocol, (2) clinical assessment which includes the pedNIHSS, (3) baseline bloods, (4) agreed on minimum imaging dataset (for MRI axial T1, T2, FLAIR, diffusion weighted imaging, apparent diffusion coefficient, susceptibility weighted imaging and time of flight MR angiography sequences and inclusion of CT angiography in CT based protocols) and (5) eligibility for thrombolysis. These elements align with the national childhood stroke guideline. There will be resource-related protocol variability in (1) type of initial imaging (CT vs MRI), (2) use of perfusion imaging and (3) ECR (case-by-case discussion for children presenting in the 6–24 hours’ time-window, and (4) whether this is performed on site at the paediatric hospital or at an adult comprehensive stroke centre.

### Study participants, inclusion and exclusion criteria

During the pre-implementation phase, from 2015 to 2017, children aged 1 month to 18 years with radiologically confirmed stroke will be retrospectively identified from ICD10 principal diagnosis codes for stroke and then medical record review.

**Figure 1:**
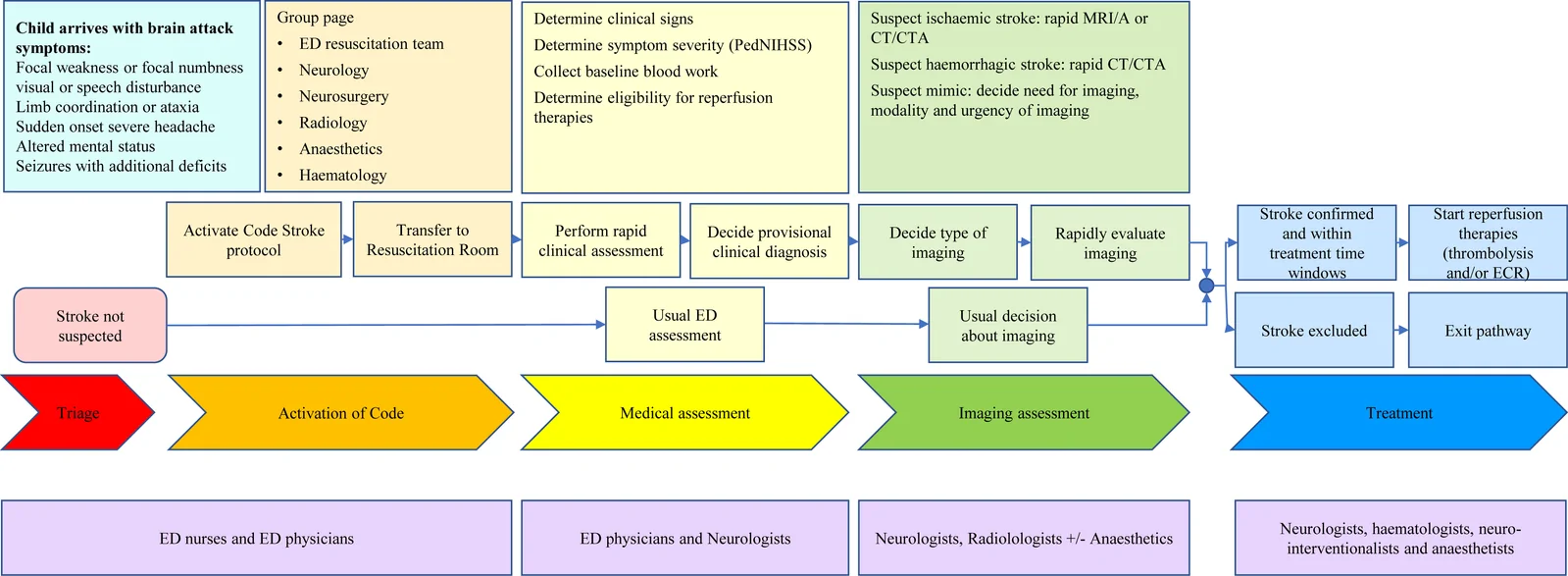
Code Stroke protocol^*^. ^*^Pathways may differ from site to site, based on already established workflow practices and available resources. CTA, CT angiography; ECR, endovascular clot retrieval; ED, emergency department; MRA, magnetic resonance angiography.

During the Code Stroke protocol development and post implementation phases from 2018 to 2026, children aged 1 month to less than 18 years of age with symptoms of less than 24 hours duration that are suspicious for stroke (also referred to as brain attacks)^34^ will be eligible for recruitment to the PACS study. Symptoms include altered consciousness; focal face arm or leg weakness; dysarthria; dysphagia; sensory disturbance; visual disturbance; limb incoordination/ataxia; visual/sensory neglect; pupillary abnormality; severe headache with associated focal neurology and seizure(s) with associated focal neurology^28^ (figure 1). Patients will be categorised into two final diagnostic groups: stroke or mimics, based on presence or absence of acute ischaemic or haemorrhagic infarction on brain imaging. Stroke will be further sub-categorised into AIS or haemorrhagic stroke (HS) as adjudicated by a paediatric radiologist. Children with acute neurological symptoms that are not relevant for stroke will be excluded from the study.

### Interventions

#### Code Stroke protocol development and implementation (all PACS sites)

A Code Stroke protocol^35 36^ is a hospital-based protocol and multidisciplinary emergency response system designed to standardise initial clinical assessment and urgent imaging of children with suspected stroke, informed by evidence-based guidelines.^28 29^ Components along the process of care, which begins on arrival in the ED, include recognition of symptoms associated with stroke^37^ at the time of triage, which leads to activation of Code. This triggers rapid clinical assessment by ED physicians. Neurologists will confirm the presence of neurological signs and assess the severity of symptoms using the paediatric adaptation of the National Institutes of Stroke Severity Scale (pedNIHSS),^38^ and in the context of ischaemic stroke, to screen children for eligibility and contraindications to reperfusion therapies, which includes collection of bloodwork. The clinicians decide the need for and type of brain imaging (CT or MRI) to confirm stroke diagnosis. Imaging leads to decisions about reperfusion therapies for ischaemic stroke or neurosurgical intervention for HS. An example of a Code Stroke pathway is presented in figure 1.

The five tertiary paediatric hospitals without established institutional Code Stroke protocols at commencement of the study will develop and implement the protocols through establishment of multidisciplinary working groups including—but not limited to—paediatric ED physicians, neurologists, neurosurgeons, radiologists, stroke research nurses, anaesthetists, intensivists, haematologists and neuro-interventional radiologists. Current practice will be evaluated through engagement of these stakeholders, and site-specific analysis of barriers and enablers to improving stroke diagnosis will be identified, drawing on previous conceptual modelling using Value Focused Process Engineering to map the process of care in children with suspected stroke.^39^

The remaining four tertiary paediatric hospitals with pre-existing Code Stroke protocols at commencement of the study (RCH, MCH, QCH and SCH) will further refine their protocols. They will partner with state ambulance services and adult comprehensive stroke centres to investigate the accuracy and clinical utility of a paediatric stroke decision support tool and automated perfusion imaging to improve diagnostic certainty and to select children most likely to benefit from reperfusion therapies. These hospitals will also function as knowledge hubs to assist the five other hospitals with development of their Code Stroke protocols.

#### Brain Attack and Stroke in Children Screen clinical decision support tool (PACS substudy)

**Figure 2:**
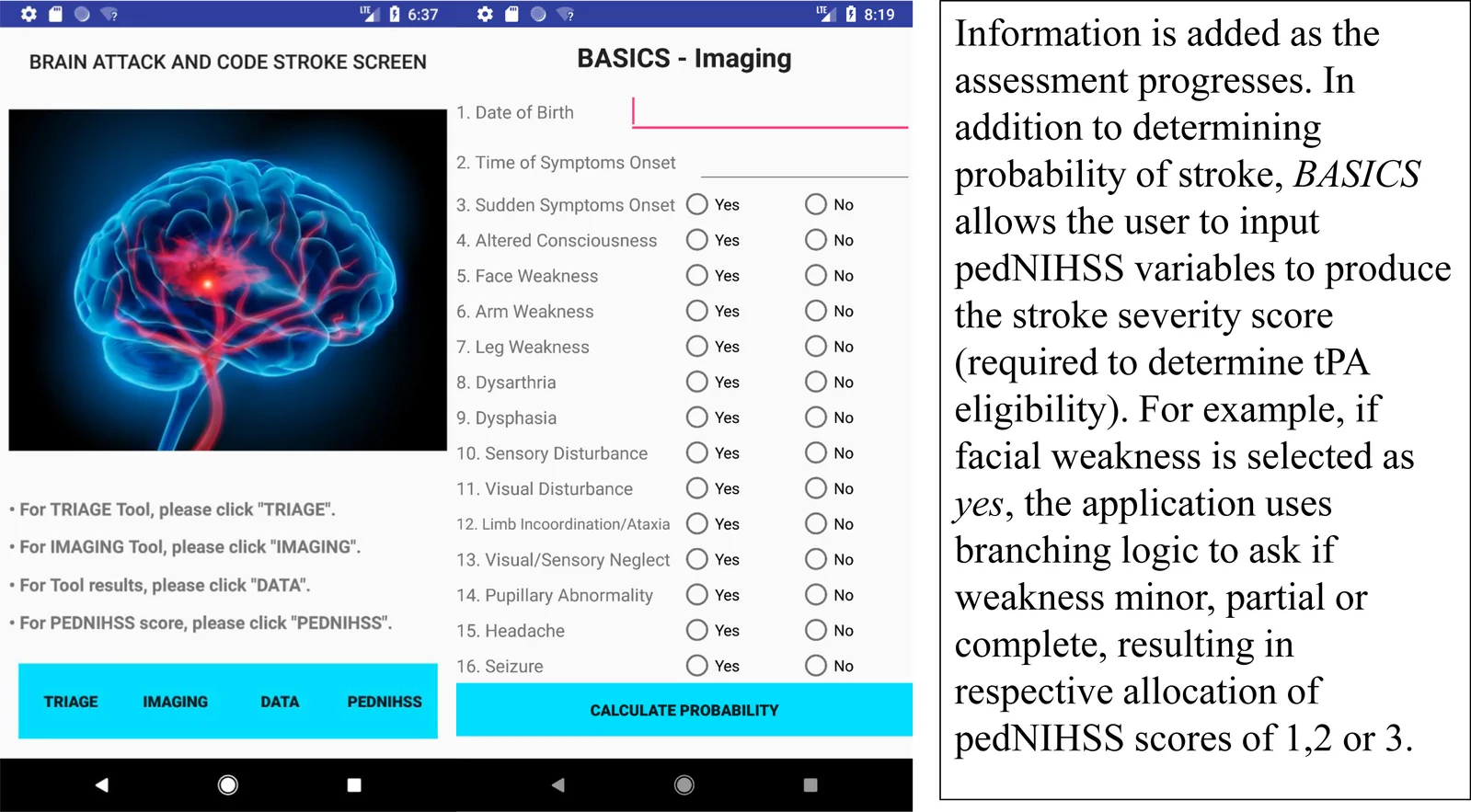
BASICS decision support tool. BASICS, Brain Attack and Stroke in Children Screen paediatric stroke recognition tool; PEDNIHSS, Paediatric National Institutes of Health Stroke Scale; tPA, tissue plasminogen activator.

The purpose of the Brain Attack and Stroke in Children Screen (BASICS) paediatric stroke recognition tool aims to increase diagnostic certainty for triage nurses and emergency physicians, using readily available demographics, process of care factors and neurological symptoms. The tool to be developed will provide the user with a probability of stroke versus mimic diagnosis and the probability of ischaemic versus HS to guide decisions about the need for Code Stroke activation and selection of CT- or MRI-based imaging. In addition to determining the probability of stroke, BASICS will allow the user to input the pedNIHSS variables to produce a stroke severity score which is essential for decisions about the use of reperfusion therapies.^28^ Unpublished pilot work using two retrospective patient cohorts suggests the tool has an overall accuracy of 84% for correct differentiation of childhood AIS from mimics and 89% for HS from mimics. A paper-based version of the BASICS decision support tool will be piloted at three hospitals (RCH, MCH and QCH). On completion of the PACS study, development of an app-based version is planned and functionality will need to be tested in a further prospective dataset (figure 2).

#### Automated perfusion-diffusion software (PACS substudy)

**Figure 3:**
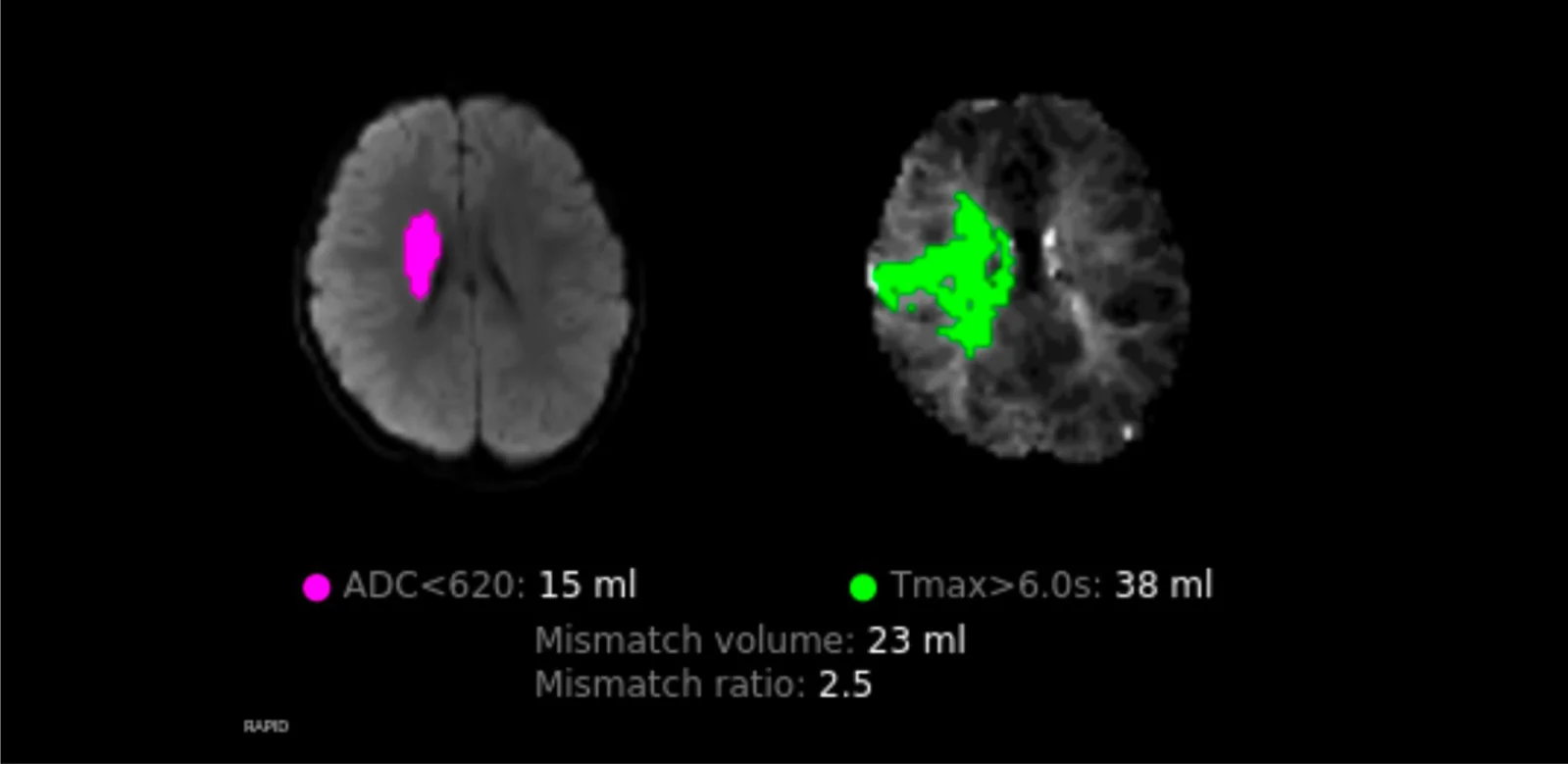
Automated perfusion diffusion software. Example of automated diffusion perfusion MRI in a child with right middle cerebral territory infarction secondary to an M1 segment middle cerebral artery vessel occlusion ADC (left) and perfusion (right) images at three 75 hours post symptom onset showing a 23 mL mismatch between the perfusion and diffusion volumes, representing salvageable brain. ADC, apparent diffusion coefficient; Tmax, time to maximum residue function.

Perfusion imaging has been demonstrated to be useful in adults to identify patients who are more likely to benefit from reperfusion therapies (eg, thrombolysis beyond 4.5 hours and ECR beyond 6 hours) by quantifying salvageable brain.^17^ PACS will investigate utility of iSchemaView RapidAI automated perfusion-diffusion software to guide decision-making for selecting children for thrombolysis and ECR. We have previously assessed the feasibility and utility of automated perfusion-diffusion imaging in a small retrospective cohort of children^19^ (figure 3).

Data are acquired with CT perfusion or MRI diffusion-weighted and perfusion imaging. Calculation of parameters related to tissue flow (perfusion) and tissue blood volume is derived and presented visually (figure 3). A central imaging repository for all PACS study de-identified, processed RAPID perfusion maps/data will be housed at the Melbourne Brain Centre at The Royal Melbourne Hospital. Utilisation of RapidAI perfusion software will not be used by all centres and will be at the discretion of the neuroradiologist and associate investigators at participating PACS study sites.

### Study procedure post-protocol implementation at participating sites

In children with suspected stroke, where symptoms are considered suspicious for stroke, the ED team will activate a Code Stroke, resulting in rapid assessment by the neurology team. During the hours 08:30—18:00 on weekdays, the triage nurse/or ED nurse caring for the patient will notify the site PACS study nurse research coordinator of eligible children. The PACS study coordinator will follow the participant during their ED stay and track their progress. For children presenting after hours and on weekends, eligible cases will be identified through (1) notifications to the study coordinators from the on-call neurology team, (2) daily review of the ED triage list to identify potentially eligible patients and (3) direct triage notifications through the electronic medical record.

In children with relevant symptoms where stroke is not suspected, the patient discharge list in the ED triage system will be reviewed daily for participants presenting outside normal working hours (or if the PACS coordinator is unavailable during hours). This will allow the identification of children who present outside recruitment hours and data will be collected retrospectively. In participants who present after hours or who are discharged from the ED without PACS study coordinator involvement, the family will be contacted by phone within 5 days of discharge to obtain consent to continue. Participants with suspected stroke who do not undergo imaging will be contacted by phone call 6 weeks from their ED presentation to ensure there has been no change in the discharge diagnosis.

## Data collection

### Pre-implementation and pre-protocol development phase

All sites will retrospectively collect data for confirmed strokes by case record review from 2015 to 2017, using the ICD-10 codes: I61.0–I61.6, I61.8, I61.9, I62.9, I63.0–I63.6, I63.8, I63.9, I64.0 and G45, for the primary aim of determining time to diagnosis prior to implementation of Code Stroke protocols across study sites (table 1).^40 41^

**Table 1:** Data to be collected

Variables	Pre-implementation (2015–2017) confirmed stroke	Post implementation phase (2018 onwards) stroke	Post implementation phase (2018 onwards) mimics
Demographics	✓	✓	✓
Prehospital care*	✓	✓	✓
Presenting clinical features	✓	✓	✓
pedNIHSS (baseline, 24 and 48 hours)	✓†	✓	✓‡
EMS call taker and paramedic coded diagnoses*	✓	✓	✓
Sensitivity of ED physician diagnosis		✓	✓
Time to diagnosis§	✓	✓	✓
Timelines of care (pre- and post-arrival)	✓	✓	✓
Length of stay, acute hospital and inpatient rehabilitation	✓	✓	✓¶
Follow-up phone call following ED discharge for mimics to confirm no change in diagnosis		✓	✓
Rates of reperfusion therapy use (t-PA and ECR)	✓	✓	
Reperfusion therapy related serious adverse events	✓	✓	
Neurological outcomes: Outcomes: (PSOM and mRS) at discharge 7, 30 and 90 days. mRS at 30 and 90 days	✓	✓	
Child Health Utility (CHU-9D)		✓	
Costs of childhood stroke, using hospital/Medicare linked services health utilisation cost data		✓	

* For the subgroups of children transported to ED by ambulance.

† Paediatric version National Institutes of Health Stroke Scale (pedNIHSS) and Paediatric Stroke Outcome Measures (PSOM) may be derived retrospectively in some (but not all) patients from clinical assessment notes and should be collected if applicable.

‡ Only at baseline.

§ Time to diagnosis will be collected for all children presenting to the emergency department, regardless of mode of arrival and for inpatients where a Code Stroke is activated.

¶ Only if admitted.

ECR, endovascular clot retrieval; ED, emergency department; EMS, emergency medical services; mRS, modified Rankin score; tPA, tissue plasminogen activator.

### Protocol development and post implementation phases

During this phase, data will be collected for children presenting to the ED with brain attack symptoms relevant for stroke.

For secondary aim (4), in participants with confirmed stroke, the health economic impact will be determined using the Child Health Utility-9D (CHU-9D) as a paediatric generic preference-based measure of health-related quality of life, to allow calculation of quality-adjusted life years (QALYs) in cost-utility analyses.^42^ Data will be entered into a REDCap database (table 1).

For secondary aim (5), the Paediatric Stroke Outcome Measures^40^ and the paediatric version of the modified Rankin Scale^41^ will be derived where possible if data are available for the retrospective patient cohort in the pre-implementation phase, and prospectively collected in the protocol development and post implementation phases.

The full list of data variables collected is detailed in online supplemental e-Table 2.

## Sample size and power calculations

We anticipate 326 patients with suspected stroke will be recruited during the protocol development and prospective phases of the study (based on numbers of patients recruited to institutional stroke registries in Victoria and New South Wales). Recent literature suggests that approximately one in three children with stroke symptoms will have a final diagnosis of stroke following imaging.^31 43–45^ Therefore, we anticipate 98 participants with a radiologically confirmed diagnosis of stroke in each study phase. Estimates of pre-implementation time to stroke diagnosis are based on extrapolation of local published data^13^ and audit data provided by three study sites. Estimates of post implementation time to stroke diagnosis are based on audit data from Queensland Children’s Hospital, where the proportion of children diagnosed within 4.5 hours increased from 15% to 40% during the 12-month period following introduction of their institutional PACS in 2019. We estimate that 15% of children will be diagnosed within 4.5 hours in the pre-implementation phase, increasing to 40% in the post implementation phase.

**Figure 4:**
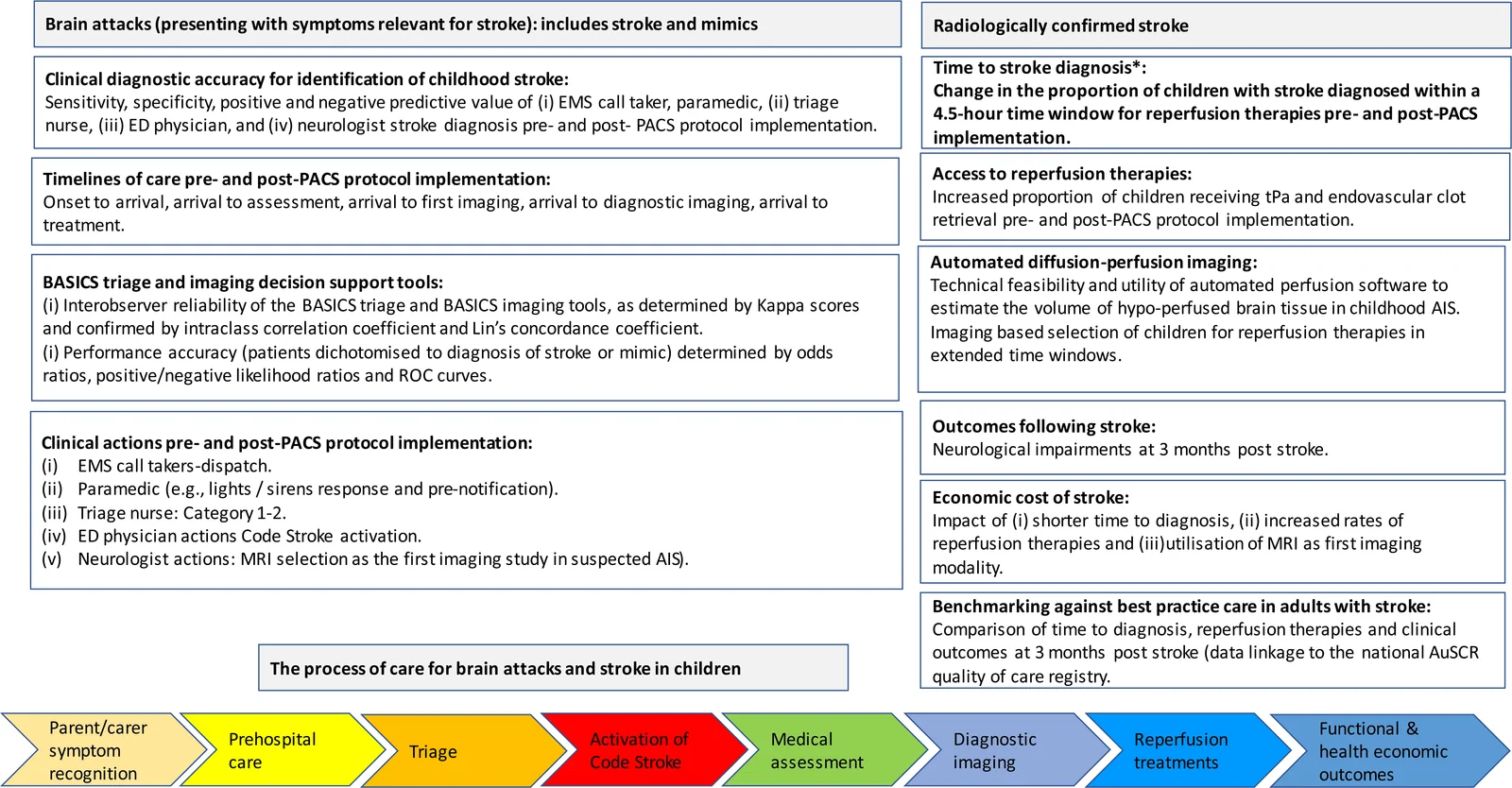
PACS study outcome measures and analyses. AIS, arterial ischaemic stroke; AuSCR, Australian Clinical Stroke Registry; BASICS, Brain Attack and Stroke in Children Screen; ED, emergency department; EMS, emergency medical services; PACS, Paediatric Acute Code Stroke; ROC, receiver operating characteristic; tPa, tissue plasminogen activator.

The *primary outcome* is the change in the proportion of children with stroke diagnosed within a 4.5-hour time window for reperfusion therapies, pre- and post-PACS implementation (figure 4). Time to diagnosis will be based on radiological confirmation of ischaemic infarction or haemorrhage on CT or MRI. This will be determined by calculating the time elapsed from last seen well to completion of neuroimaging, which is electronically recorded on imaging studies.

Due to the hospital-based nature of the proposed intervention, when powering this study, we have taken appropriate care of the hierarchical nature of the data, where individual patients are clustered within the treating hospitals. It is therefore appropriate to anticipate the presence of a design effect (expressed through the value of the intraclass correlation coefficient), as would have to be done in the situation of cluster-randomised studies. In addition, different hospitals will be able to recruit different numbers of patients, thus there will be a variation of cluster sizes.

Nine hospitals are participating in the study. At the time of receiving grant funding in July 2020, seven hospitals had agreed to participate in the study. Based on this consideration, assuming the settings of alpha=0.05, the intraclass correlation coefficient of 0.05 (corresponding to a small-to-medium hospital-specific effect) and the coefficient of variation of hospital-based cluster sizes of 0.5 (medium-to-high level of variation) with at least seven hospitals, recruiting the 98 patients with radiologically confirmed diagnosis of stroke in the pre-PACS implementation phase and 98 patients in the post-PACS protocol implementation stage (on average 14 patients per hospital for both pre-implementation and post implementation stages), will yield 80% power to detect a change from 0.15 to 0.40 in proportion of children diagnosed within 4.5 hours of symptom onset.

In addition, this sample size will be sufficient for further validation and continuous updating of the BASICS tool in order to further improve its predictive capacity (as the total number of screened patients is expected to be at least three times the number of confirmed strokes).^30 31^

## Data analysis plan

### Outcomes

The *primary outcome* is the change in the proportion of children with stroke diagnosed within a 4.5-hour time window for reperfusion therapies, pre- and post-PACS implementation. This will be estimated using a mixed-effect logistic regression model with the dichotomous variable ‘diagnosed within 4.5-hour time window (yes/no)’ as the dependent variable, implementation phase as an independent fixed effect and hospitals as random effects.

*Secondary outcomes* (figure 4) include:

- The proportion of children receiving reperfusion therapies will be analysed as for the primary outcome.
- Accuracy of emergency medical services (EMS) call taker, paramedic and ED physician stroke diagnosis against the gold standard diagnosis of radiologically confirmed stroke. Change in correctness of stroke (vs mimic) diagnosis, pre-implementation and post implementation of PACS will be estimated using a random-effect logistic regression model with dichotomised correctness of diagnosis as the dependent variable, intervention phase as an independent variable and hospital as a random effect.
- The impact of EMS call taker, paramedic and ED physician actions (eg, lights/sirens response and prenotification, PACS activation, MRI selection as the first imaging study in suspected AIS) on time to diagnosis will be analysed. Differences in times will be analysed using clustered quartile regression, with hospitals as clusters.
- The economic impact of shorter time to diagnosis, increased utilisation of MRI imaging and increased rates of reperfusion therapy in stroke patients will be measured using the CHU-9D, and hospitals linked services health utilisation and cost data.

Markov cohort model will be developed to quantify per-capita, national healthcare and societal costs economic costs, and the associated lifetime loss of QALYs and life years.

- Performance accuracy of the BASICS tools will be evaluated (patients dichotomised to diagnosis of stroke or mimic) by ORs, positive/negative likelihood ratios and receiver operating characteristic curves.
- Interobserver reliability of the BASICS tools will be evaluated using kappa score and confirmed by intraclass correlation coefficient and Lin’s concordance coefficient.
- Benchmarking primary and secondary outcomes (1) and (2) against adult best practice care through data linkage to the Australian Clinical Stroke Registry (AuSCR)^46^ and ambulance services data. Descriptive statistics will be used to summarise characteristics of children treated in paediatric versus adult hospitals, with differences tested using χ^2^ tests for categorical variables and rank-sum test for non-parametric continuous variables.

## Dissemination

### Study oversight

Governance for the PACS Study will be provided by Stroke Foundation, Melbourne, Australia. The PACS Study CI will report the study progress to the Chief Executive Officer and Executive Director Stroke Programmes, Research and Innovation of Stroke Foundation.

### Study timeline, ethics approval and dissemination plan

Study commencement at recruiting sites was delayed by the COVID-19 pandemic. Ethics approval was granted to commence the study at the coordinating site on 5 August 2021. Site approval was granted from August 2021 until September 2023 across the eight participating sites. Data collection will continue until mid-2026.

Study findings will be disseminated through presentations at major stroke scientific conferences and in peer-reviewed journals, following final approval by all contributing authors.

## Discussion

More than half of children who experience a stroke have long-term impairments impacting motor, cognitive, language, behaviour and social functions.^7^ The overall goal of the PACS study is to improve survival outcomes for Australian and New Zealand children with stroke through the development and implementation of childhood Code Stroke protocols, targeting key steps along the process of care.^39^ The primary objective is to bring childhood stroke diagnosis into the 4.5-hour time-window for reperfusion therapies and, by extension, to narrow the gap between adults and children in accessing life-changing treatments.

There is limited reporting of the efficacy and safety of reperfusion therapies in children because randomised controlled trials have excluded patients <18 years^5 6^ and stand-alone paediatric trials are not thought to be feasible following the failed international TIPS trial.^47^ Consensus-based childhood stroke guidelines allow for reperfusion therapies but there is a strong imperative to collect real-world safety and efficacy data for these interventions.^28 29^

The diagnostic utility of automated perfusion imaging to quantify brain at risk of infarction will be prospectively evaluated in the PACS study, which is aligned with the shift in adults towards adopting tissue-based (volume of brain as quantified by perfusion imaging), rather than time-based decisions about use of reperfusion therapies. This approach is more scientifically valid in children, given the more varied causes, which differ from adults^48^ and delays to stroke diagnosis.

Testing the utility of clinical decision support tools will inform paramedic clinical practice guidelines. The PACS study will also, for the first time, generate a national Australian childhood stroke dataset demonstrating the impact of reperfusion therapies on functional outcomes, which will be benchmarked against adults with stroke using the AuSCR registry.

The health economic analyses will generate data about the economic burden of childhood stroke and the associated loss of QALYs in Australian children, to allow more targeted use of health resources. Knowledge gains from the study will be incorporated in the Stroke Foundation of Australia living guidelines for stroke.^49^

Limitations include bias related to the mixed retrospective and prospective data collection. Different hospitals will be able to recruit different numbers of patients, thus there will be variation in cluster sizes. Protocols are being developed and tested in tertiary hospitals but approximately 50% of children with stroke initially present to non-tertiary paediatric hospitals.^9^ Therefore, an important priority for future research is to adapt Code Stroke protocols developed in the PACS study to rural and regional centres. Stroke telemedicine will be an important component of this initiative.^50^ These future approaches will ensure that all children with stroke, no matter where they reside or to which health setting they present, will have access to coordinated systems of acute stroke care, to technologies that facilitate rapid diagnosis and to reperfusion therapies that limit the extent of brain injury.

## Supplementary material

10.1136/bmjno-2026-001582 online supplemental file 1

## Data Availability

Data sharing not applicable as no datasets were generated and/or analysed for this study.
