# Supplementary material for: Australian Paediatric Acute Code Stroke study: study protocol for a quasi-experimental trial measuring the impact of Code Stroke protocols on time to diagnosis and access to reperfusion therapies
Source: BMJ Neurol Open. 2026 Sep 3;8(2):e001582. doi: 10.1136/bmjno-2026-001582 (PMC13548288; doi:10.1136/bmjno-2026-001582)
Supplement: Supplementary file 1 [file bmjno-8-2-s001.docx]

**Supplementary e-Table 1:** Study Sites

| **Paediatric primary stroke centres** | **Adult comprehensive stroke centres** |  |
| --- | --- | --- |
| ***Group 1 Centres yet to establish Code Stroke Protocols*** | |  |
| Children’s Hospital at Westmead, Sydney, NSW | Westmead Hospital |  |
| John Hunter Children’s Hospital, Newcastle, NSW | John Hunter Hospital˄ |  |
| Women’s & Children’s Hospital, Adelaide, SA | Royal Adelaide Hospital |  |
| Perth Children’s Hospital, Perth, WA | Sir Charles Gairdner Hospital |  |
| Starship Children’s Hospital, Auckland, NZ | Auckland City Hospital˄ |  |
| ***Group 2 Centres with established Code Stroke Protocols*** | |  |
| Royal Children’s Hospital, Melbourne, VIC* | Royal Melbourne Hospital |  |
| Queensland Children’s Hospital, Brisbane, QLD* | Royal Brisbane and Women’s Hospital |  |
| Monash Children’s Hospital, Melbourne, VIC | Monash Medical Centre^ |  |
| Sydney Children’s Hospital, Sydney, NSW | Prince of Wales Hospital^ |  |

*Knowledge hubs with established PACS. ^Co-located with paediatric hospitals.

NSW: New South Wales, SA: South Australia, WA: Western Australia, NZ: New Zealand, QLD: Queensland, VIC: Victoria.

**Supplementary e-Table 2**: Full list of study variables

| **Variable** | **Details** |
| --- | --- |
| Record ID Number | Numeric |
| Data collection type | 1, Retrospective \| 2, Prospective |
| Hospital | 1, Royal Children's Hospital \| 2, Monash Children's Hospital \| 3, Children's Hospital at Westmead \| 4, Sydney Children's Hospital, Randwick \| 5, John Hunter Children's Hospital \| 6, Queensland Children's Hospital \| 7, Women's and Children's Hospital, Adelaide \| 8, Perth Children's Hospital \| 9, Starship Children's Hospital \| 10, Tasmanian Health Service \| 11, Northern Territory Health Service |
| **Demographics and past history** |  |
| Date of Birth | Date |
| Sex | 1, Female \| 2, Male \| 3, Another term (please specify) |
| Country of birth | 1, Australia \| 2, New Zealand \| 3, Other (please specify) |
| Aboriginal or Torres Straight Islander | 1, Aboriginal but not Torres Strait Islander origin \| 2, Torres Strait islander but not Aboriginal origin \| 3, Both Aboriginal and Torres Strait Islander origin \| 4, Neither Aboriginal or Torres Strait Islander \| 5, Indigenous not otherwise described \| 6, Missing/Not stated |
| Ethnicity (for NZ participants) | 1, New Zealand European \| 2, Maori \|3, Samoan \| 4, Tongan \| 5, Niuean \| 6, Chinese \|7, Indian \| 8, Other (such as Dutch, Japanese, Tokelauen) - Please specify |
| Past medical history relevant for stroke | Yes or No |
| Past medical history details | 1, Cardiac disease \| 2, Anaemia \| 3, Malignancy \| 4, Autoimmune disorder \| 5, Head or neck trauma \| 6, None \| 7, Bilateral arteriopathy (Moyamoya disease) |
| Recent history of infection | 1, Yes \| 0, No |
| Previous stroke | 1, Yes \| 0, No |
| **Timelines pre-hospital arrival** |  |
| Onset date/time | Time |
| Time last seen well if wake up stroke | Time |
| Ambulance called | 1, Yes \| 0, No |
| Triple zero call date | Date |
| Triple zero call time | Time |
| Ambulance dispatch date | Date |
| Ambulance dispatch time | Time |
| Ambulance arrival at scene time | Time |
| Ambulance departure from scene time | Time |
| Ambulance off stretcher time | Time |
| **Paramedic and ED care** |  |
| EMS call taker problem stated | Free text |
| EMS call taker triage category | Numeric 1-5 |
| EMS coding | Numeric |
| EMS Location | 1, VIC \| 2, SA \| 3, NSW \| 4, QLD \| 5, WA \| 6, NZ \| 7, NT \| 8, TAS |
| EMS call taker diagnosis | 1, Stroke \| 2, Mimic \| 3, Other |
| Paramedic diagnosis | 1, Stroke \| 2, Mimic \| 3, Other |
| Paramedic prenotification | 1, Yes \| 0, No |
| Lights and sirens | 1, Yes \| 0, No |
| Ambulance type | 1, Mobile Stroke Unit (MSU) \| 2, MICA \| 3, Other |
| Was this ED based at a: | 1, PACS Study Site \| 2, Metropolitan Hospital \| 3, Regional Hospital \| 4, Rural Hospital |
| Date of ED arrival | Date |
| Time of ED arrival | Time |
| Time seen by triage nurse | Time |
| Time seen by doctor | Time |
| Time seen by Dr (if inpatient stroke) | Time |
| **Process of care/Mode of presentation** |  |
| Referral by health professional | 1, Yes \| 0, No |
| Transport by ambulance | 1, Yes \| 0, No |
| Triage category | Numeric 1-5 |
| Presenting problem | Presenting problem (HAS triage diagnostic coding) |
| Description of presenting problem | Free text |
| Location at stroke event | 1, Home \| 2, School \| 3, Other. \| 3, Other hospital |
| In hospital stroke (current inpatient) | 1, Yes \| 0, No |
| Patient presenting to another hospital with stroke symptoms (prior to transfer to PACS site) | 1, Yes \| 0, No |
| Was any imaging performed at the peripheral hospital? | 1, Yes \| 0, No |
| Date and time patient transferred out of peripheral hospital | Date and time |
| Inter hospital transfer | 1, Yes \| 0, No |
| Reason for transfer | 1, Need for IV tPA \| 2, Need for Stroke Unit Care \| 3, Need for Rehabilitation \| 4, Need for Brain Imaging only \| 5, Need for ICU \| 6, Need for specialist medical assessments \| 7, Need for surgical interventions \| 8, Need for diagnostic tests \| 9, Need for coordinated care by Stroke Service \| 10, Need for endovascular therapy \| 11, Unknown \|12, Other |
| **Symptom time course** |  |
| Sudden onset of symptoms | 1, Yes \| 0, No |
| Woke with symptoms | 1, Yes \| 0, No |
| Unknown symptom onset time | 1, Yes \| 0, No |
| Symptoms improving | 1, Yes \| 0, No |
| Well in the week before presentation | 1, Yes \| 0, No |
| **Symptoms** |  |
| Headache | 1, Yes \| 0, No |
| Vomiting | 1, Yes \| 0, No |
| Dizziness | 1, Yes \| 0, No |
| Speech disturbance | 1, Yes \| 0, No |
| Focal numbness | 1, Yes \| 0, No |
| Focal weakness | 1, Yes \| 0, No |
| Seizure | 1, Yes \| 0, No |
| Altered mental status | 1, Yes \| 0, No |
| Loss of consciousness | 1, Yes \| 0, No |
| Visual disturbance | 1, Yes \| 0, No |
| Ataxia | 1, Yes \| 0, No |
| Vertigo | 1, Yes \| 0, No |
| Photophobia | 1, Yes \| 0, No |
| Phonophobia | 1, Yes \| 0, No |
| Other neurological symptoms | 1, Yes \| 0, No |
| Other neurological symptoms | 1, Yes \| 0, No |
| Symptoms unknown | 1, Yes \| 0, No |
| **General signs** |  |
| Systolic blood pressure | Numeric |
| Diastolic blood pressure | Numeric |
| Heart rate | Numeric |
| Saturations | Numeric |
| Temperature (degrees) | Numeric |
| Glucose level measured | 1, Yes \| 0, No |
| Glucose value | Numeric |
| Abnormal pupillary response | 1, Yes \| 0, No |
| **GCS** |  |
| GCS Eye opening | 1, None \| 2, To pain \| 3, To verbal command \| 4, Spontaneous |
| GCS Verbal response | 1, No response \| 2, Incomprehensible sounds \| 3, Inappropriate words \| 4, Confused \| 5, Oriented, appropriate |
| GCS Motor response | 1, None \| 2, Abnormal extension to pain (decerebrate) \| 3, Abnormal flexion to pain (decorticate) \| 4, Withdraws to pain \| 5, Localizes to pain \| 6, Obeys commands |
| **NIHSS scale** |  |
| Baseline PedNIHSS available? | 1, Yes \| 0, No |
| Was the patient intubated? | 1, Yes \| 0, No |
| Total NIHSS Score at baseline | Numeric (range 0-42) |
| PEDNIHSS level of consciousness | 0, Alert; keenly responsive \|1, Not alert, but arousable by stimulation to obey, answer, or respond \| 2, Not alert, requires repeated stimulation, or is obtunded and requires strong/painful stimulation to make movements (not stereotyped) \| 3, Responds only with reflex motor or autonomic effects or totally unresponsive, flaccid, areflexic |
| PEDNIHSS level of conscious questions | 0, Answers both questions correctly \| 1, Answers one question correctly \| 2, Answers neither question correctly |
| PEDNIHSS level of conscious commands | 0, Performs both tasks correctly \| 1, Performs one task correctly \| 2, Performs neither task correctly |
| PEDNIHSS abnormal eye movements (gaze palsy) | 0, Normal \| 1, Partial gaze (abnormal gaze in one or both eyes but no forced deviation or total gaze paresis \| 2, Forced deviation or total gaze paresis not overcome by oculocephalic manoeuvre |
| PEDNIHSS visual field defect | 0, No visual loss \| 1, Partial hemianopia \| 2, Complete hemianopia \| 3, Bilateral hemianopia (blind including cortical blindness) |
| PEDNIHSS facial weakness | 0, Normal symmetrical movement \| 1, Minor paralysis (flattened nasolabial fold, asymmetry on smiling) \| 2, Partial paralysis (total or near total paralysis of lower face) \| 3, Complete paralysis of one or both sides (absence of facial movement in the upper and lower face) |
| PEDNIHSS left arm weakness | 0, No drift, limb holds 90 (or 45) degrees for full 10 seconds \| 1, Drift, limb holds 90 (or 45) degrees, but drifts down before full 10 seconds; does not hit bed or other support \| 2, Some effort against gravity, limb cannot get to/or maintain (if cued) 90 (or 45) degrees, drifts down to bed, but has some effort against gravity \| 3, No effort against gravity, limb falls \| 4, No movement \| 9, Amputation, joint fusion |
| PEDNIHSS right arm weakness | 0, No drift, limb holds 90 (or 45) degrees for full 10 seconds \| 1, Drift, limb holds 90 (or 45) degrees, but drifts down before full 10 seconds; does not hit bed or other support \| 2, Some effort against gravity, limb cannot get to/or maintain (if cued) 90 (or 45) degrees, drifts down to bed, but has some effort against gravity \| 3, No effort against gravity, limb falls \| 4, No movement \| 9, Amputation, joint fusion |
| PEDNIHSS left leg weakness | 0, No drift, leg holds 30 degrees position for full 5 seconds \| 1, Drift, leg falls by the end of the 5 second period but does not hit bed \| 2, Leg falls to bed by 5 seconds, but has some effort against gravity \| 3, No effort against gravity, leg falls to be immediately \| 4, No movement \| 9, Amputation, joint fusion |
| PEDNIHSS right leg weakness | 0, No drift, leg holds 30 degrees position for full 5 seconds \| 1, Drift, leg falls by the end of the 5 second period but does not hit bed \| 2, Leg falls to bed by 5 seconds, but has some effort against gravity \| 3, No effort against gravity, leg falls to be immediately \| 4, No movement \| 9, Amputation, joint fusion |
| PEDNIHSS ataxia | 0, Absent \| 1, Present in one limb \| 2, Present in two limbs \| 9, Amputation, joint fusion |
| PEDNIHSS sensory disturbance | 0, Normal; no sensory loss \| 1, Mild to moderate sensory loss; patient feels pinprick is less sharp or dull on the affected side, or there is a loss of superficial pain with pinprick but patient is aware he/she is being touched \| 2, Severe to total sensory loss; patient is not aware of being touched in the face, arm and leg |
| PEDNIHSS best language | 0, No aphasia, normal \| 1, Mild to moderate aphasia \| 2, Severe aphasia \| 3, Mute, global aphasia; no usable speech or auditory comprehension |
| PEDNIHSS dysarthria | 0, Normal \| 1, Mild to moderate; patient slurs at least some words and, at worst, can be understood with some difficulty \| 2, Severe; patient's speech is so slurred as to be unintelligible in the absence of or out of proportion to any dysphasia, or is mute/anarthric \| 9, Intubated or other physical barrier |
| PEDNNIHSS sensory or visual neglect | 0, No abnormality \| 1, Visual, tactile, auditory, spatial, or personal inattention or extinction to bilateral simultaneous stimulation in one of the sensory modalities \| 2, Profound hemi-inattention or hemi-inattention to >1 modality; does not recognize on hand or orients to only one side of space |
| PEDNIHSS total score at baseline | Numeric (range 0-42) |
| **Additional neurological signs** |  |
| Hand weakness | 1, Yes \| 0, No |
| Altered arm sensation | 1, Yes \| 0, No |
| Altered leg sensation | 1, Yes \| 0, No |
| Altered facial sensation | 1, Yes \| 0, No |
| Sensory neglect | 1, Yes \| 0, No |
| Unable to walk | 1, Yes \| 0, No |
| Other neurological signs | 1, Yes \| 0, No |
| Other neurological signs (free text) | Free text |
| Unable to assess (infant) | 1, Yes \| 0, No |
| **Actions** |  |
| Telestroke consultation | 1, Yes \| 0, No |
| Code stroke activation | 1, Yes \| 0, No |
| Time of Code Stroke Activation | Time |
| Neurology consultation in the ED | 1, Yes \| 0, No |
| Neurosurgery consultation in the ED | 1, Yes \| 0, No |
| **Imaging** |  |
| Brain imaging performed | 1, Yes \| 0, No |
| Was initial imaging performed at another hospital prior to patient transfer? | 1, Yes \| 0, No |
| Upload imaging to Central Imaging Repository | 1, Yes \| 0, No |
| Brain CT scan performed | 1, Yes \| 0, No |
| CT date | Date |
| CT time | Time |
| CT result | CT result |
| CT angiogram performed | 1, Yes \| 0, No |
| CT angiogram date & time | Date & time |
| CT angiogram result | 1, Occlusion \| 2, Stenosis \| 0, Normal \| 3, Arteriovenous Malformation \| 4, AV Fistula \| 5, Aneurysm \| 6, Cavernoma \| 7, Tumour \| 8, CSVT \| 9, Other |
| CT angiogram occlusion site | 1, ICA \| 2, M1 \| 3, M2 \| 4, M3 \| 5, ACA \| 6, PCA \| 7, BA \| 8, VA \| 9, Other \| 0, Not occluded |
| CT perfusion imaging performed | Yes or No |
| Date and time of CT perfusion scan | Date and time |
| CT core volume: mls | Numeric |
| CT perfusion volume: mls | Numeric |
| CT mismatch volume: mls | Numeric |
| CT mismatch ratio: numerical | Numeric |
| Brain MRI performed | Yes or No |
| MRI date | Date |
| MRI time | Time |
| MRI result | 1, Stroke \| 2, Other abnormal \| 0, Normal |
| MR angiogram performed | 1, Yes \| 0, No |
| MR angiogram date & time | Date and time |
| MR angiogram result | 1, Occlusion \| 2, Stenosis \| 0, Normal \| 3, Arteriovenous Malformation (AVM) \| 4, AV Fistula \| 5, Aneurysm \| 6, Cavernoma \| 7, Tumour \| 8, CSVT \| 9, Other |
| MR angiogram occlusion site | 1, ICA \| 2, M1 \| 3, M2 \| 4, M3 \| 5, ACA \| 6, PCA \| 7, BA \| 8, VA \| 9, Other \| 0, Not occluded |
| MR perfusion imaging performed | 1, Yes \| 0, No |
| Date and time of MR perfusion scan | Date and time of MR perfusion scan |
| MR core volume (mls) | Numeric |
| MR perfusion volume (mls) | Numeric |
| MR mismatch volume (mls) | Numeric |
| MR mismatch ratio (mls) | Numeric |
| Type of perfusion imaging software used | 1, RAPID \| 2, MiSTAR \| 3, None |
| Was the initial scan diagnostic? | 1, Yes \| 0, No |
| Date and time of diagnostic scan | Date and time |
| Modality of diagnostic scan | 1, MRI \| 2, CT |
| Was a cerebral angiogram performed? | Yes or No |
| Data and time of cerebral angiogram | Data and time |
| Was neck imaging performed | 1, Yes \| 0, No |
| What modality of neck imaging was performed | 1, Ultrasound \| 2, CTA \| 3, MRA |
| **Diagnosis** |  |
| ED physician clinical diagnosis | 1, Stroke \| 2, Mimic \| 3, Inpatient (not done) |
| ED diagnostic certainty | Numeric (range 0-100%) |
| Neurologist clinical diagnosis | 1, Stroke \| 2, Mimic |
| Neurologist diagnostic certainty | Numeric (range 0-100%) |
| Final diagnosis | 1, Stroke \| 2, Migraine \| 3, Seizure \| 4, Encephalitis/cerebritis \| 5, Meningitis \| 6, Tumour (includes CNS and haematological) \| 7, Metabolic encephalopathy \| 8, Other encephalopathy (free text) \| 9, ADEM/brain demyelination \| 10, Myelitis Gullain-Barre \| 11, Bells Palsy \| 12, Cerebellitis \| 13, Peripheral neuropathy \| 14, Conversion disorder \| 15, Drug intoxication \| 16, Syncope \| 17, Other (free text) |
| Final diagnosis (other) | Free text |
| Did the patient's diagnosis change at 6 weeks | Yes or No |
| What was the final diagnosis | 1, Stroke \| 2, Migraine \| 3, Seizure \| 4, Encephalitis/cerebritis \| 5, Meningitis \| 6, Tumour (includes CNS and haematological) \| 7, Metabolic encephalopathy \| 8, Other encephalopathy (free text) \| 9, ADEM/brain demyelination \| 10, Myelitis Gullain-Barre \| 11, Bells Palsy \| 12, Cerebellitis \| 13, Peripheral neuropathy \| 14, Conversion disorder \| 15, Drug intoxication \| 16, Syncope \| 17, Other (free text) |
| 6-week final diagnosis (other) | Free text |
| If stroke, what was the stroke subtype | 1, Ischaemic \| 2, Haemorrhagic |
| **Stroke diagnosis** |  |
| Stroke subtype | 1, Ischaemic \| 2, Haemorrhagic (Intracerebral haemorrhage) \| 3, Cerebral Sinovenous Thrombosis (CSVT) \| 4, TIA |
| Circulation | 1, Anterior circulation \| 2, Posterior circulation \| 3, Both \| 4, Not applicable |
| Ischaemic stroke mechanism | 1, Small vessel arteriopathy \| 2, Focal cerebral arteriopathy \| 3, Bilateral cerebral arteriopathy \| 4, Aortic/cervical arteriopathy, \| 5, Cardioembolic \| 6, Multifactorial \| 7, Other \| 8, Unknown \| 9, Not Applicable |
| Haemorrhagic stroke mechanism | 1, AVM \| 2, Aneurysm \| 3, Cavernoma \| 4, Tumour \| 5, Other \| 6, Unknown \| 7, Not applicable \| 8, AV Fistula \| 9, CSVT |
| **Stroke treatments** |  |
| tPA treatment | 1, Yes \| 0, No |
| tPA agent | 1, Alteplase \| 2, Tenecteplase |
| Date tPA treatment | Date |
| Time tPA treatment | Time |
| Endovascular clot retrieval | 1, Yes \| 0, No |
| Date endovascular clot retrieval | Date |
| Time endovascular clot retrieval groin puncture | Time |
| Time endovascular clot retrieval completion of procedure | Time |
| Recanalisation | 1, Yes \| 0, No |
| TICI reperfusion | 1, TICI 0 complete occlusion, no perfusion \| 2, TICI 1 Penetration with minimal perfusion \| 3, TICI 2a Partial perfusion - only partial filling (less than two-thirds) of the entire vascular territory is visualised \| 4, TICI 2b Partial perfusion - complete filling of all the expected vascular territory is visualised but the filling is slower than normal \| 5, TICI 3 Complete perfusion |
| Decompressive Craniotomy | 1, Yes \| 0, No |
| Clot Evacuation | 1, Yes \| 0, No |
| External ventricular drain or ventriculostomy | 1, Yes \| 0, No |
| Insertion of ICP monitor | 1, Yes \| 0, No |
| AVM Resection | 1, Yes \| 0, No |
| Endovascular AVM Coiling | 1, Yes \| 0, No |
| Endovascular AVM Embolization | 1, Yes \| 0, No |
| Endovascular AVM Stenting | 1, Yes \| 0, No |
| Endovascular AVM Vessel Sacrifice | 1, Yes \| 0, No |
| AVM Stereotactic Radiosurgery | 1, Yes \| 0, No |
| Clipping of cerebral aneurysm | 1, Yes \| 0, No |
| Infarct volume at 24 hours (mls) | Numeric |
| Did the patient receive LMW Heparin (as an acute treatment) | 1, Yes \| 0, No |
| **Stroke treatment complications** |  |
| Symptomatic ICH at 24 hours | 1, Yes \| 0, No |
| ICH detail | 0, No haemorrhage detected \| 1, I: Haemorrhagic infarct 1: small petechiae, <50% of infarct area (includes laminar necrosis) \| 2, II: haemorrhagic infarct 2: confluent petechiae, >50% of infarct area \| 3, III: Parenchyma haemorrhage 1: blood <30% of infarct area, no or slight space effect \| 4, IV: Parenchyma haemorrhage 1: blood >30% of infarct area, substantial space effect |
| Extracranial haemorrhage | 1, Yes \| 0, No |
| Recurrent ischaemic stroke | 1, Yes \| 0, No |
| Recurrent stroke of unknown type | 1, Yes \| 0, No |
| Malignant MCA infarction | 1, Yes \| 0, No |
| Malignant Cerebral Oedema | 1, Yes \| 0, No |
| Neurological deterioration attributed to swelling of index stroke | 1, Yes \| 0, No |
| Neurological deterioration NOT attributed to swelling of index stroke | 1, Yes \| 0, No |
| Asymptomatic intracranial haemorrhage identified by routine imaging | 1, Yes \| 0, No |
| Angioedema | 1, Yes \| 0, No |
| Allergic Reaction | 1, Yes \| 0, No |
| Gastrointestinal Bleeding | 1, Yes \| 0, No |
| Vasospasm | 1, Yes \| 0, No |
| Dissection | 1, Yes \| 0, No |
| Groin Haematoma | 1, Yes \| 0, No |
| Wire Perforation | 1, Yes \| 0, No |
| Embolization into another vascular territory | 1, Yes \| 0, No |
| Residual AVM lesion | 1, Yes \| 0, No |
| Post operative haemorrhage | 1, Yes \| 0, No |
| Hydrocephalus | 1, Yes \| 0, No |
| Distal embolism or infarction | 1, Yes \| 0, No |
| Other serious adverse event | 1, Yes \| 0, No |
| Other serious adverse event | Free text |
| **Outcome 24 hours** |  |
| 24-hour PedNIHSS available? | 1, Yes \| 0, No |
| Was the patient intubated? | 1, Yes \| 0, No |
| Total NIHSS Score at 24 hours | Numeric (range 0-42) |
| PEDNIHSS level of consciousness | 0, Alert; keenly responsive \|1, Not alert, but arousable by stimulation to obey, answer, or respond \| 2, Not alert, requires repeated stimulation, or is obtunded and requires strong/painful stimulation to make movements (not stereotyped) \| 3, Responds only with reflex motor or autonomic effects or totally unresponsive, flaccid, areflexic |
| PEDNIHSS level of conscious questions | 0, Answers both questions correctly \| 1, Answers one question correctly \| 2, Answers neither question correctly |
| PEDNIHSS level of conscious commands | 0, Performs both tasks correctly \| 1, Performs one task correctly \| 2, Performs neither task correctly |
| PEDNIHSS abnormal eye movements (gaze palsy) | 0, Normal \| 1, Partial gaze (abnormal gaze in one or both eyes but no forced deviation or total gaze paresis \| 2, Forced deviation or total gaze paresis not overcome by oculocephalic manoeuvre |
| PEDNIHSS visual field defect | 0, No visual loss \| 1, Partial hemianopia \| 2, Complete hemianopia \| 3, Bilateral hemianopia (blind including cortical blindness) |
| PEDNIHSS facial weakness | 0, Normal symmetrical movement \| 1, Minor paralysis (flattened nasolabial fold, asymmetry on smiling) \| 2, Partial paralysis (total or near total paralysis of lower face) \| 3, Complete paralysis of one or both sides (absence of facial movement in the upper and lower face) |
| PEDNIHSS left arm weakness | 0, No drift, limb holds 90 (or 45) degrees for full 10 seconds \| 1, Drift, limb holds 90 (or 45) degrees, but drifts down before full 10 seconds; does not hit bed or other support \| 2, Some effort against gravity, limb cannot get to/or maintain (if cued) 90 (or 45) degrees, drifts down to bed, but has some effort against gravity \| 3, No effort against gravity, limb falls \| 4, No movement \| 9, Amputation, joint fusion |
| PEDNIHSS right arm weakness | 0, No drift, limb holds 90 (or 45) degrees for full 10 seconds \| 1, Drift, limb holds 90 (or 45) degrees, but drifts down before full 10 seconds; does not hit bed or other support \| 2, Some effort against gravity, limb cannot get to/or maintain (if cued) 90 (or 45) degrees, drifts down to bed, but has some effort against gravity \| 3, No effort against gravity, limb falls \| 4, No movement \| 9, Amputation, joint fusion |
| PEDNIHSS left leg weakness | 0, No drift, leg holds 30 degrees position for full 5 seconds \| 1, Drift, leg falls by the end of the 5 second period but does not hit bed \| 2, Leg falls to bed by 5 seconds, but has some effort against gravity \| 3, No effort against gravity, leg falls to be immediately \| 4, No movement \| 9, Amputation, joint fusion |
| PEDNIHSS right leg weakness | 0, No drift, leg holds 30 degrees position for full 5 seconds \| 1, Drift, leg falls by the end of the 5 second period but does not hit bed \| 2, Leg falls to bed by 5 seconds, but has some effort against gravity \| 3, No effort against gravity, leg falls to be immediately \| 4, No movement \| 9, Amputation, joint fusion |
| PEDNIHSS ataxia | 0, Absent \| 1, Present in one limb \| 2, Present in two limbs \| 9, Amputation, joint fusion |
| PEDNIHSS sensory disturbance | 0, Normal; no sensory loss \| 1, Mild to moderate sensory loss; patient feels pinprick is less sharp or dull on the affected side, or there is a loss of superficial pain with pinprick but patient is aware he/she is being touched \| 2, Severe to total sensory loss; patient is not aware of being touched in the face, arm and leg |
| PEDNIHSS best language | 0, No aphasia, normal \| 1, Mild to moderate aphasia \| 2, Severe aphasia \| 3, Mute, global aphasia; no usable speech or auditory comprehension |
| PEDNIHSS dysarthria | 0, Normal \| 1, Mild to moderate; patient slurs at least some words and, at worst, can be understood with some difficulty \| 2, Severe; patient's speech is so slurred as to be unintelligible in the absence of or out of proportion to any dysphasia, or is mute/anarthric \| 9, Intubated or other physical barrier |
| PEDNNIHSS sensory or visual neglect | 1, Yes \| 0, No |
| Was 24-hour imaging performed? | 1, Yes \| 0, No |
| Upload imaging to Central Imaging Repository | 1, Yes \| 0, No |
| **Outcome 48 hours** |  |
| 48-hour PedNIHSS available? | 1, Yes \| 0, No |
| Was the patient intubated? | 1, Yes \| 0, No |
| Total NIHSS Score at 48 hours | Numeric (range 0-42) |
| PEDNIHSS level of consciousness | 0, Alert; keenly responsive \|1, Not alert, but arousable by stimulation to obey, answer, or respond \| 2, Not alert, requires repeated stimulation, or is obtunded and requires strong/painful stimulation to make movements (not stereotyped) \| 3, Responds only with reflex motor or autonomic effects or totally unresponsive, flaccid, areflexic |
| PEDNIHSS level of conscious questions | 0, Answers both questions correctly \| 1, Answers one question correctly \| 2, Answers neither question correctly |
| PEDNIHSS level of conscious commands | 0, Performs both tasks correctly \| 1, Performs one task correctly \| 2, Performs neither task correctly |
| PEDNIHSS abnormal eye movements (gaze palsy) | 0, Normal \| 1, Partial gaze (abnormal gaze in one or both eyes but no forced deviation or total gaze paresis \| 2, Forced deviation or total gaze paresis not overcome by oculocephalic manoeuvre |
| PEDNIHSS visual field defect | 0, No visual loss \| 1, Partial hemianopia \| 2, Complete hemianopia \| 3, Bilateral hemianopia (blind including cortical blindness) |
| PEDNIHSS facial weakness | 0, Normal symmetrical movement \| 1, Minor paralysis (flattened nasolabial fold, asymmetry on smiling) \| 2, Partial paralysis (total or near total paralysis of lower face) \| 3, Complete paralysis of one or both sides (absence of facial movement in the upper and lower face) |
| PEDNIHSS left arm weakness | 0, No drift, limb holds 90 (or 45) degrees for full 10 seconds \| 1, Drift, limb holds 90 (or 45) degrees, but drifts down before full 10 seconds; does not hit bed or other support \| 2, Some effort against gravity, limb cannot get to/or maintain (if cued) 90 (or 45) degrees, drifts down to bed, but has some effort against gravity \| 3, No effort against gravity, limb falls \| 4, No movement \| 9, Amputation, joint fusion |
| PEDNIHSS right arm weakness | 0, No drift, limb holds 90 (or 45) degrees for full 10 seconds \| 1, Drift, limb holds 90 (or 45) degrees, but drifts down before full 10 seconds; does not hit bed or other support \| 2, Some effort against gravity, limb cannot get to/or maintain (if cued) 90 (or 45) degrees, drifts down to bed, but has some effort against gravity \| 3, No effort against gravity, limb falls \| 4, No movement \| 9, Amputation, joint fusion |
| PEDNIHSS left leg weakness | 0, No drift, leg holds 30 degrees position for full 5 seconds \| 1, Drift, leg falls by the end of the 5 second period but does not hit bed \| 2, Leg falls to bed by 5 seconds, but has some effort against gravity \| 3, No effort against gravity, leg falls to be immediately \| 4, No movement \| 9, Amputation, joint fusion |
| PEDNIHSS right leg weakness | 0, No drift, leg holds 30 degrees position for full 5 seconds \| 1, Drift, leg falls by the end of the 5 second period but does not hit bed \| 2, Leg falls to bed by 5 seconds, but has some effort against gravity \| 3, No effort against gravity, leg falls to be immediately \| 4, No movement \| 9, Amputation, joint fusion |
| PEDNIHSS ataxia | 0, Absent \| 1, Present in one limb \| 2, Present in two limbs \| 9, Amputation, joint fusion |
| PEDNIHSS sensory disturbance | 0, Normal; no sensory loss \| 1, Mild to moderate sensory loss; patient feels pinprick is less sharp or dull on the affected side, or there is a loss of superficial pain with pinprick but patient is aware he/she is being touched \| 2, Severe to total sensory loss; patient is not aware of being touched in the face, arm and leg |
| PEDNIHSS best language | 0, No aphasia, normal \| 1, Mild to moderate aphasia \| 2, Severe aphasia \| 3, Mute, global aphasia; no usable speech or auditory comprehension |
| PEDNIHSS dysarthria | 0, Normal \| 1, Mild to moderate; patient slurs at least some words and, at worst, can be understood with some difficulty \| 2, Severe; patient's speech is so slurred as to be unintelligible in the absence of or out of proportion to any dysphasia, or is mute/anarthric \| 9, Intubated or other physical barrier |
| PEDNNIHSS sensory or visual neglect | 0, No abnormality \| 1, Visual, tactile, auditory, spatial, or personal inattention or extinction to bilateral simultaneous stimulation in one of the sensory modalities \| 2, Profound hemi-inattention or hemi-inattention to >1 modality; does not recognize on hand or orients to only one side of space |
| Other SAE | 1, Yes \| 0, No |
| Recurrent ischaemic stroke | 1, Yes \| 0, No |
| Recurrent stroke of unknown type | 1, Yes \| 0, No |
| Malignant MCA infarction | 1, Yes \| 0, No |
| Neurological deterioration attributed to swelling of index stroke | 1, Yes \| 0, No |
| Neurological deterioration NOT attributed to swelling of index stroke | 1, Yes \| 0, No |
| Asymptomatic intracranial haemorrhage identified by routine imaging | 1, Yes \| 0, No |
| **Outcome day seven** |  |
| PSOM right sensorimotor 7 days | 9, Not done \| 0, None \| 0.5, Mild but no impact on function \| 1, Moderate with some functional limitations \| 2, Severe or profound with missing function |
| PSOM left sensorimotor 7 days | 9, Not done \| 0, None \| 0.5, Mild but no impact on function \| 1, Moderate with some functional limitations \| 2, Severe or profound with missing function |
| PSOM expressive language 7 days | 9, Not done \| 0, None \| 0.5, Mild but no impact on function \| 1, Moderate with some functional limitations \| 2, Severe or profound with missing function |
| PSOM receptive language 7 days | 9, Not done \| 0, None \| 0.5, Mild but no impact on function \| 1, Moderate with some functional limitations \| 2, Severe or profound with missing function |
| PSOM cognitive/behavioural 7 days | 9, Not done \| 0, None \| 0.5, Mild but no impact on function \| 1, Moderate with some functional limitations \| 2, Severe or profound with missing function |
| PSOM total score 7 days | Numeric (0-10) |
| Modified Rankin Scale | 0, No symptoms \| 1, No significant disability. Able to carry out all usual activities, despite some symptoms \| 2, Slight disability. Able to look after own affairs without assistance, but unable to carry out all previous activities \| 3, Moderate disability. Requires some help, but able to walk unassisted \| 4, Moderately severe disability. Unable to walk and attend to bodily needs without assistance \| 5, Severe disability. Bedridden, incontinent and requiring constant nursing care and attention \| 6, Dead \| 7, Not Done |
| Total Modified Rankin Scale Score | Numeric (range 0-6) |
| Has any imaging been performed since admission? | 1, Yes \| 0, No |
| Imaging uploaded to central repository | 1, Yes \| 0, No |
| **Discharge** |  |
| Medication at discharge | 1, Antiplatelet \| 2, Anticoagulant \| 3, Combination \| 4, None |
| Date of discharge from hospital | Date |
| Did the patient require inpatient rehabilitation | 1, Yes \| 0, No |
| Date of transfer to inpatient rehabilitation unit | Date |
| Date of discharge from inpatient rehabilitation unit | Date |
| Patient deceased during admission. | 1, Yes \| 0, No |
| Date of death | Date |
| Death due to stroke | 1, Yes \| 0, No |
| **Outcome day 30** |  |
| Lost to follow-up | 1, Yes \| 0, No |
| PSOM right sensorimotor 30 days | 9, Not done \| 0, None \| 0.5, Mild but no impact on function \| 1, Moderate with some functional limitations \| 2, Severe or profound with missing function |
| PSOM left sensorimotor 30 days | 9, Not done \| 0, None \| 0.5, Mild but no impact on function \| 1, Moderate with some functional limitations \| 2, Severe or profound with missing function |
| PSOM expressive language 30 days | 9, Not done \| 0, None \| 0.5, Mild but no impact on function \| 1, Moderate with some functional limitations \| 2, Severe or profound with missing function |
| PSOM receptive language 30 days | 9, Not done \| 0, None \| 0.5, Mild but no impact on function \| 1, Moderate with some functional limitations \| 2, Severe or profound with missing function |
| PSOM cognitive/behavioural 30 days | 9, Not done \| 0, None \| 0.5, Mild but no impact on function \| 1, Moderate with some functional limitations \| 2, Severe or profound with missing function |
| PSOM total score 30 days | Numeric (range 0-10) |
| **Surveillance Imaging Day 90** |  |
| Has any imaging follow-up imaging been performed since patient's last scan? | 1, Yes \| 0, No |
| Imaging uploaded to central repository | 1, Yes \| 0, No |
| Brain MRI performed | 1, Yes \| 0, No |
| MRI date | Date |
| MRI time | Time |
| MRI result | 1, Stroke \| 2, Other abnormal \| 0, Normal |
| MR angiogram performed | 1, Yes \| 0, No |
| MR angiogram result | 1, Occlusion \| 2, Stenosis \| 0, Normal |
| MR angiogram occlusion site | 1, ICA \| 2, M1 \| 3, M2 \| 4, M3 \| 5, ACA \| 6, PCA \| 7, BA \| 8, VA \| 9, Other \| 0, Not occluded |
| Imaging uploaded to central repository | 1, Yes \| 0, No |
| Ischaemic stroke mechanism | 1, Small vessel arteriopathy \| 2, Focal cerebral arteriopathy \| 3, Bilateral cerebral arteriopathy \| 4, Aortic/cervical arteriopathy, \| 5, Cardioembolic \| 6, Multifactorial \| 7, Other \| 8, Unknown \| 9, Not Applicable |
| Haemorrhagic stroke mechanism | 1, AVM \| 2, Aneurysm \| 3, Cavernoma \| 4, Tumour bleed \| 5, Other \| 6, Unknown \| 7, Not applicable |
| Epilepsy | 1, Yes \| 0, No |
| **Outcome day 90** |  |
| Lost to follow-up | 1, Yes \| 0, No |
| PSOM right sensorimotor 90 days | 9, Not done \| 0, None \| 0.5, Mild but no impact on function \| 1, Moderate with some functional limitations \| 2, Severe or profound with missing function |
| PSOM left sensorimotor 90 days | 9, Not done \| 0, None \| 0.5, Mild but no impact on function \| 1, Moderate with some functional limitations \| 2, Severe or profound with missing function |
| PSOM expressive language 90 days | 9, Not done \| 0, None \| 0.5, Mild but no impact on function \| 1, Moderate with some functional limitations \| 2, Severe or profound with missing function |
| PSOM receptive language 90 days | 9, Not done \| 0, None \| 0.5, Mild but no impact on function \| 1, Moderate with some functional limitations \| 2, Severe or profound with missing function |
| PSOM cognitive/behavioural 90 days | 9, Not done \| 0, None \| 0.5, Mild but no impact on function \| 1, Moderate with some functional limitations \| 2, Severe or profound with missing function |
| PSOM total score 90 days | Numeric (range 0-10) |
| Modified Rankin Scale | 0, No symptoms \| 1, No significant disability. Able to carry out all usual activities, despite some symptoms \| 2, Slight disability. Able to look after own affairs without assistance, but unable to carry out all previous activities \| 3, Moderate disability. Requires some help, but able to walk unassisted \| 4, Moderately severe disability. Unable to walk and attend to bodily needs without assistance \| 5, Severe disability. Bedridden, incontinent and requiring constant nursing care and attention \| 6, Dead \| 7, Not Done |
| Total Modified Rankin Scale Score | Numeric (range 0-6) |
| Death at 90 days | 1, Yes \| 0, No |
| Date of death | Date |
| Death due to stroke | 1, Yes \| 0, No |
| Recurrence at 90 days | 1, Yes \| 0, No |
| **CHUD-9** |  |
| CHUD-9 report completed | 1, Yes \| 0, No |
| CHUD-9 patient report | Date and time |
| CHUD-9 patient report | Today I feel worried so I will tick this box |
| CHUD-9 patient report | Today I feel sad so I will tick this box |
| CHUD-9 patient report | Today I feel pain so I will tick this box |
| CHUD-9 patient report | Today I feel tired so I will tick this box |
| CHUD-9 patient report | Today I feel annoyed so I will tick this box |
| CHUD-9 patient report | School work / Homework |
| CHUD-9 patient report | Last night I had trouble sleeping so I will tick this box |
| CHUD-9 patient report | Today I had problems with my daily routine so I will tick this box |
| CHUD-9 patient report | Today I was able to join in activities so I will tick this box |
| CHUD-9 parent report | Date and time |
| CHUD-9 parent report | Today my child feels worried so I will tick this box |
| CHUD-9 parent report | Today my child feels sad so I will tick this box |
| CHUD-9 parent report | Today my child feels pain so I will tick this box |
| CHUD-9 parent report | Today my child feels tired so I will tick this box |
| CHUD-9 parent report | Today my child feels annoyed so I will tick this box |
| CHUD-9 parent report | School work / Homework |
| CHUD-9 parent report | Today my child had trouble sleeping so I will tick this box |
| CHUD-9 parent report | Today my child had trouble with their daily routine so I will tick this box |
| CHUD-9 parent report | Today my child feels activities so I will tick this box |

**Supplementary e-Figure 1: Study consent process**
